# A lethal incident during an intergroup encounter in bonobos

**DOI:** 10.1038/s41598-026-40297-w

**Published:** 2026-03-23

**Authors:** Miguel Gareta García, Lillian J. Fornof, Kris H. Sabbi, Floris Martin, Eliana Sonderling, Juliet De Rozario, Mina Cikara, Martin Surbeck

**Affiliations:** 1 Department of Human Evolutionary Biology, Harvard University, Cambridge, 02138 USA; 2 Department of Psychology, Harvard University, Cambridge, MA USA; 3 Department of Human Behavior, Ecology and Culture, Max Planck Institute for Evolutionary Anthropology, Leipzig, Germany

**Keywords:** Bonobo, Community, Intergroup encounter, Aggression, Out-group, Ecology, Ecology, Evolution, Psychology, Psychology, Zoology

## Abstract

Although neighbouring bonobo communities often display tolerance and cooperation when associating together, aggression is also commonly observed during intergroup encounters. Here, we describe an observation at the Kokolopori research site (DRC) in which a coalition of individuals from the neighbouring Kokoalongo community aggressed an adult female from the Ekalakala community. This happened during an ongoing intergroup encounter that had begun earlier as the two communities travelled and foraged together. Shortly after the attack, the 52-day-old infant of the attacked female was carried by two immatures of Kokoalongo. The infant showed signs of distress before being taken by an adult Kokoalongo female, the mother of the immatures. The infant died the following day, and the adult female continued to carry the corpse for two additional days. This unique case of infant acquisition by unrelated out-group individuals immediately following aggression against the mother underscores that bonobo intergroup encounters can have lethal outcomes. These events highlight the complexity and unpredictability of intergroup dynamics in this species.

**Supplementary Information:**

The online version contains supplementary material available at 10.1038/s41598-026-40297-w.

## Introduction

Non-human primates provide key insights into the evolutionary roots of human intergroup relations, ranging from tolerance to aggression and, in some species, lethal violence. As in humans, non-human primate conflicts can involve lethal aggression toward out-group individuals, including infants^1–10^. Our closest relatives, chimpanzees and bonobos, exhibit a marked contrast in the prevalence and expression of intergroup aggression and infanticide. Chimpanzees (*Pan troglodytes*) have been observed, albeit infrequently, to engage in lethal aggression against out-group individuals and commit infanticide both within and between groups^1,2,4–9,11^. In contrast, bonobos (*Pan paniscus*) lack clear evidence of either behaviour (but see^12^) and often associate with out-group individuals for extended periods^13,14^. While this divergence offers important insight into the ancestral state of intergroup dynamics around the human-Pan split, it does not imply that bonobo intergroup tolerance is uniform. Rather, occasional intense aggression occurs in bonobos but is rarer than in chimpanzees.

Both bonobos and chimpanzees live in multi-male, multi-female communities, where females generally disperse from their natal groups around the time that they reach sexual maturity^15–17^(see exceptions in both bonobos^12^and chimpanzees^18,19^). Despite male-biased sexual dimorphism in both species, bonobo females—unlike their chimpanzee counterparts—can attain high social status within their groups^20,21^and do not experience sexual coercion by males^22–24^. While intergroup interactions in chimpanzees are predominantly hostile, frequently leading to aggression and involving territorial defence, bonobos are known for their tolerant intergroup encounters (henceforth referred to as encounters) that are more frequent when food is abundant^14,25–27^. During encounters, individuals from neighbouring communities affiliate and engage in cooperative behaviours such as food sharing and grooming^13,14,27–29^, even as competition over food resources intensifies in this context. Still, despite decades of wild bonobo research in different study sites, lethal intergroup aggression or infanticide has not yet been described in bonobos, even though aggressive behaviours are common during encounters and sometimes result in physical injuries and elevated stress responses^30,31^. Such observations underscore the complex and nuanced nature of bonobo intergroup dynamics^13,32,33^.

We describe an intergroup encounter during which Kokoalongo individuals attacked the Ekalakala female Rose, seized her 52-day-old infant Rouille, and carried her until the infant’s death the following day. Chapman, an adult Kokoalongo female, continued carrying the body for two additional days. We summarise the incident and discuss its implications for in-group-out-group biases and coalitionary aggression during encounters.

## Results

### Description of the events

The described observations began on August 6, 2024, during an encounter between the Kokoalongo (total group size: 38 individuals) and Ekalakala (22 individuals) bonobo communities at the Kokolopori Bonobo Reserve in the Democratic Republic of Congo (see demographics in Table S1). During this encounter, members of the Kokoalongo community directed coalitionary aggression against Rose, an adult female from Ekalakala in the lower third of her community’s hierarchy (see dominance hierarchies in Table S2), after which her young female infant, Rouille, was seized and carried by Kokoalongo individuals. On that day, we observed several members of Kokoalongo’s “C” family handle Rouille, including a 6-year-old female juvenile (Curtis) and her 12-year-old adolescent male sibling (Cobain), before the infant was ultimately carried by their mother, Chapman, who is in the top quartile of her community’s hierarchy (Table S2). After Rouille’s death the following day, Chapman continued carrying her body for two additional days. Throughout the episode, Rouille was never returned to her mother, and Rose was not observed making any attempts to reclaim her infant from any of the carriers. In the weeks and months preceding this event, the two communities had already engaged in several intergroup encounters, consistent with the regular pattern observed previously (see Fig. S1, Table S3). Below, we provide a detailed account of these interactions, including Kokoalongo individuals’ behaviour toward Rouille, Rose’s responses, the events leading to Rouille’s death, and Chapman’s subsequent corpse-carrying behaviour.

On August 6, 2024, at 09:00, an adult male bonobo, Zappa, from the Kokoalongo community vocalised from a fruiting *Landolphia* sp. vine, a seasonally important food source for bonobos, while several other individuals foraged nearby for underground fungi. Zappa’s vocalization may have attracted other individuals, as a few minutes later, some individuals arrived at the same tree. These individuals included Kokoalongo adult females (Chapman, Oliday, Gloria, PJ, and Tyler), Ekalakala adult females (Ivoire, Azur, and Rose), Ekalakala adult males (Gris and Noir), and immigrant females Celeste and Nancy.

At 10:55, the observers documented an initial aggressive incident, although the canopy obstructed their view. Immediately thereafter, a coalition comprising the adult Kokoalongo male Zappa and four adult Kokoalongo females (Chapman, Oliday, PJ, and Tyler) chased the Ekalakala female Rose through the canopy. Rose descended to the ground, where the Kokoalongo coalition physically attacked her. Observers then lost sight of Rose and her aggressors as she fled after the attack. At 13:30, Rose reappeared without her infant and behaved normally, moving between the ground and canopy while feeding intermittently. Only a few days later did observers notice what appeared to be several day-old genital wounds (Fig. S2), although her behaviour at the time showed no signs of acute injury.

**Fig. 1:**
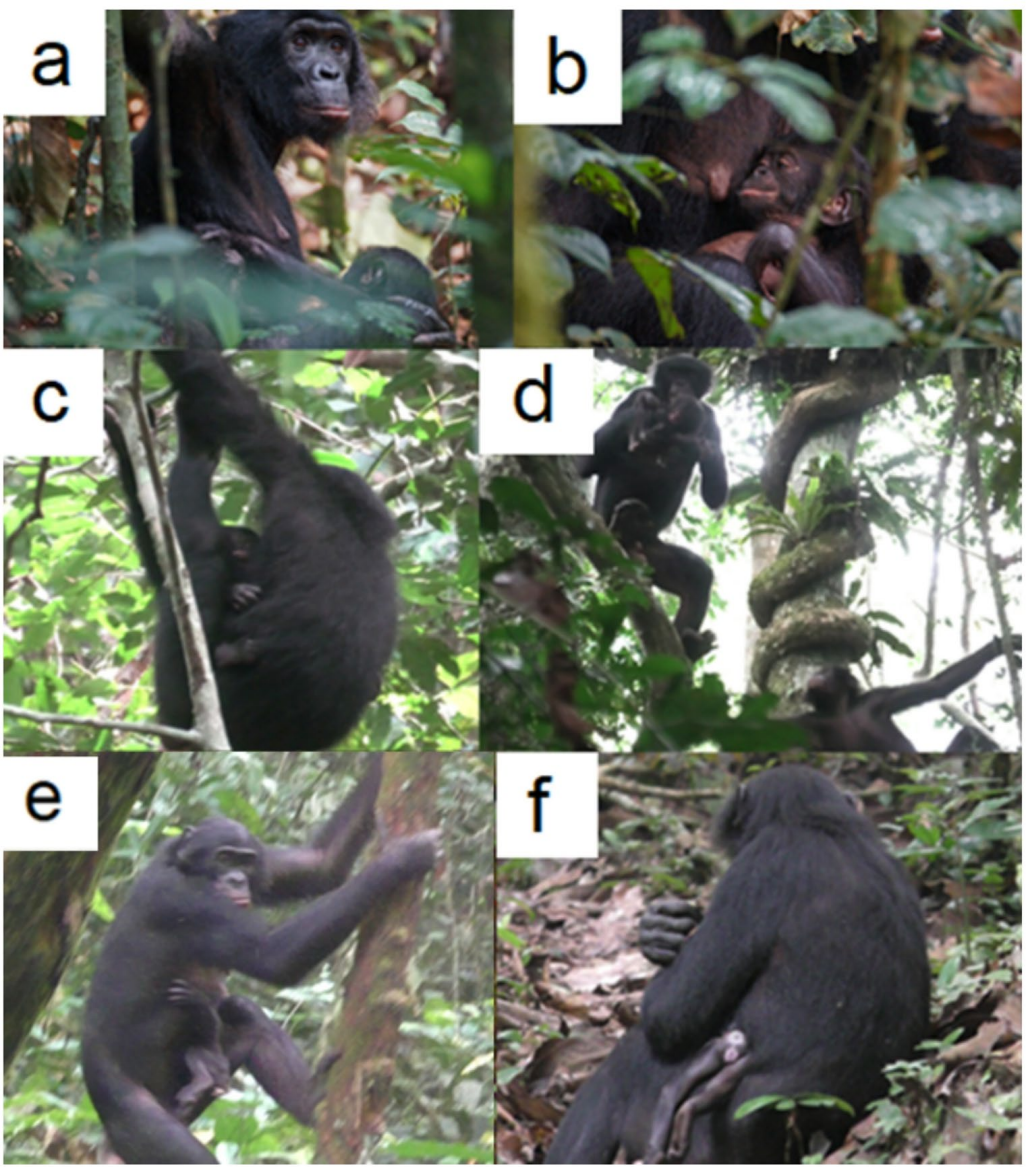
Photos of Rouille: (**a**,**b**) Rose holding her infant on July 13 and July 31, 2024 (LF). (**c**) Curtis holding Rouille on August 6, 2024, at 11:47 (FM). (**d**) Cobain carrying Rouille, followed by Curtis, at 11:55 (FM). (**e**) Chapman climbing down a tree, carrying Rouille with her left thigh while alive, on August 8, 2024 (MGG). (**f**) Chapman holding Rouille, deceased, on August 8, 2024 (MGG).

At 11:10, just 15 min after the initial aggression, the juvenile female Curtis was seen carrying Rouille, Rose’s 52-day-old daughter, while her adolescent brother, Cobain, followed closely (Fig. 1b). Curtis moved through the trees with Rouille held against her abdomen (Fig. 1c,d), and Rouille displayed no apparent signs of distress, either behavioural or vocal. Throughout this period, Rouille clung to Curtis during locomotion and had no visible injuries (see Video 1).

At 13:48, Cobain forcibly took Rouille from Curtis, carrying the infant upside down, provoking loud, continuous screams. Their mother, Chapman, began to follow Cobain as he moved. At 13:53, Cobain paused to sniff Rouille, who continued to scream. He manipulated the infant and inspected her anogenital area, still holding her upside-down. Moments later, Cobain dropped Rouille from a height of approximately three meters and quickly descended to retrieve her, with Curtis closely following. After regaining possession, Cobain swung Rouille by the arm.

Between 14:01 and 14:06, Cobain dragged Rouille along the ground before climbing into a tree, where he pressed her head down with one foot. While moving along the branches, he intermittently lifted her upside down and occasionally swung her back and forth. At 14:06, Rouille fell about five meters to the ground, but Curtis immediately retrieved her and resumed ventral carrying. The observers lost sight of Curtis and Rouille until 14:12.

At 14:12, Chapman was observed carrying Rouille ventrally, with Curtis riding dorsally and Cobain following. Although Rouille clung to Chapman’s abdomen, Chapman provided additional support with one hand while moving on the ground and used her thighs to stabilise Rouille during arboreal travel (Fig. 1e).

Throughout this time, the encounter between Ekalakala and Kokolongo continued, and observers followed both groups, recording encounter dynamics, including social interactions and 30-minute scans of which bonobos were present (party composition). Between 11:00 and 14:30, observers tracking the Ekalakala community recorded Rose being present in the group without Chapman in the same party composition at 13:30, 15:00, and 16:00 (Supplementary Fig. S3a, Table S4). Simultaneously, observers following the Kokoalongo community documented Chapman throughout most 30-min party composition scans (Supplementary Fig. S3b). Both teams confirmed parties with members of both communities, thus being in an intergroup encounter context, and again at 16:30, shortly before the two groups nested near each other. During this time, Chapman continued to carry Rouille—who did not vocalise or show behavioural distress—until she built her evening nest at 17:00, at which point observations concluded. Altogether, Rose and Chapman were observed in the same party composition during the half-hour of the aggression (11:00–11:30) and at several subsequent times: 12:00, 12:30, 14:00, 14:30, and shortly before nesting at 16:30 and 17:00. After August 6, they were not observed together in the same party on any of the following days.

On August 7, both groups descended from their nests around 05:30. At 05:57, Chapman was observed moving on the ground, holding Rouille against her abdomen with one arm, closely followed by Curtis. At 06:00, the Ekalakala and Kokoalongo communities separated (Supplementary Fig. S3a,b, Table S4). Later, at 07:36, Chapman was seen feeding on a fruiting tree, while still carrying Rouille, and at 09:47 she foraged *on subterranean* fungi, digging with her right hand and supporting Rouille with her left.

Around 08:30 and again at 10:00, Chapman moved Rouille’s head toward her nipples, although no suckling was observed. Given that Curtis is 6 years old, Chapman may no longer be lactating, or if so, may not be producing enough milk to support a young infant^34^. While Rouille initially clung tightly to Chapman, by 10:17 her limbs hung loosely. The team of Ekalakala observers documented a brief encounter between Ekalakala and Kokoalongo members between 12:30 and 14:30; however, Rose and Chapman were never seen together in the same party during this period (Supplementary Fig. S3a,b, Table S4). At 14:15, Rouille appeared lethargic—still breathing, with her eyes partially open—but remained motionless when Chapman paused on the ground and held her on her lap (Video 2).

By 14:45, Rouille´s death was confirmed, as she was motionless and not breathing, and her limbs hung limp whenever Chapman moved. No external traumatic injuries consistent with a lethal attack (such as head trauma or obvious fractures) were visible on the corpse. The only visible lesion we documented was a small wound on the left hand (Fig. S4). Chapman continued to hold the infant against her abdomen with her left hand while moving quadrupedally. Over the next 2 h, she ate various fruits while continuing to carry Rouille ventrally. At 16:19, she was observed, for the first time, holding Rouille with her arm to her side rather than against her abdomen. Chapman built an evening nest around 17:00, still holding the deceased infant. For the following 2 days, Chapman continued to carry Rouille’s body.

August 8. Aside from interactions involving Rouille’s corpse being carried by Chapman throughout the day, Kokoalongo’s activity on August 8 consisted mainly of routine foraging and resting, with no unusual social dynamics observed. By 05:00, Rouille’s body condition and odour had deteriorated noticeably. Chapman continued to carry the corpse ventrally against her chest, supported by her left arm as she moved quadrupedally. Around 10:00, flies began to gather on the corpse when Chapman was stationary, and she occasionally swatted them away. At 10:33, while Chapman was eating some fruit on the ground, she was approached by the Kokoalongo adult female, Gloria, and by Curtis. Curtis moved within arm’s reach, touched Rouille’s swollen navel, and licked a viscous substance seeping from the corpse. Tango, the infant male of Tyler (Kokoalongo), approached and made body contact with Curtis. Curtis pushed Tango away and continued to sniff and lick Rouille’s body. During the day, whenever Chapman rested or foraged on the ground, she kept Rouille’s corpse on her lap (Fig. 1f, Video 4). At 17:11, Chapman built her evening nest, still holding Rouille’s corpse.

August 9. At 05:52, Chapman carried Rouille’s corpse by the neck with her left arm while climbing or during terrestrial movement. At 05:56, Curtis approached, inspected Rouille’s body, poked it with her finger, and licked it. At 12:18, Chapman carried Rouille while feeding on fallen fruit on the ground. At 13:32, Chapman was observed without Rouille’s corpse for the first time since August 6. Shortly thereafter, a research team located Rouille’s body.

## Discussion

The observations reported here represent the first documented case of an infant fatality resulting from the separation of a female and her infant, Rouille, after suffering an out-group coalition of five adult females and a male during an intergroup encounter. Notably, this event does not conform to established patterns of coalitionary killing, intergroup aggression, infanticide, or kidnapping reported in other non-human primates. Instead, the case entails behaviours resembling out-group infant adoption described previously in bonobos^35^ with some hallmarks of coalitionary aggression observed in other primate species^9,36^. The aggression unfolded in the canopy of a fruiting tree, a context in which intense within- and between-group feeding competition is commonplace. This ecological context likely contributed to escalation, even though the exact proximal cause cannot be definitively identified. We note that this observation is based on a single event, and that conclusions should be interpreted cautiously. We contextualise our observations amongst relevant concepts below.

During intergroup encounters, male chimpanzees often conduct coalitionary attacks—a form of collective aggression that exploits power asymmetries between aggressors and victims^37–39^. Agonistic coalitions that target members of neighbouring groups can sometimes result in lethal outcomes^2,5,8,9,40,41^. While adult male bonobos at another research site have been reported to form coalitions and be more often victims of aggressive intergroup interactions than adult females^31^, this pattern is not observed at Kokolopori, where females exhibit higher coalitionary involvement in the presence of out-group individuals^42^. In this context, it is noteworthy that the coalition described here comprised four females (Chapman, Oliday, PJ, and Tyler) and one male (Zappa), with all four females occupying the upper half of the dominance hierarchy. In contrast, the male was positioned near the lower end. This highlights the potential role of female dominance in the shaping of inter-sex coalitions against out-group individuals.

The coalitionary aggression directed at Rose prior to her infant’s loss resembles coordinated intergroup attacks reported in other primates – including chimpanzees^5,41,43,44^, mountain gorillas (*Gorilla beringei beringei*)^45^, spider monkeys (*Ateles geoffroyi*)^46^, white-handed gibbons (*Hylobates lar*)^43^and crested macaques (*Macaca nigra*)^47^– in which multiple individuals jointly targeted and physically assaulted a single out-group victim. Yet, in the case we report, the attacking coalition primarily consisted of females, and no individual directly targeted the infant. While female participation in lethal intergroup aggression has been described for chimpanzees, they typically form a minority of attacking parties^8,9,48^. Lethal attacks by all- or majority-female coalitions in primates have only been documented in female-philopatric Samango monkeys (*Cercopithecus albogularis*)^49^. Remarkably, the lethal outcome we report here appears to be a by-product rather than the primary goal of coalitionary aggression. Thus, this case differs substantially from classical intergroup lethal violence.

Infanticide, a form of lethal aggression in which the killing of an individual enhances the killer’s success due to intraspecific competition^50^, is widespread among social mammals^50–54^. In non-human primates, infanticide has been reported in many species^54,55^, including Hanuman langurs (*Semnopithecus entellus*)^56–58^, ursine colobus (*Colobus vellerosus*)^59^, capuchin monkeys (*Sapajus libidinosus*)^60–62^, chacma baboons (*Papio ursinus*)^63^, orangutans (*Pongo pygmaeus*)^64^, mountain gorillas^65^, and chimpanzees^3,6,7,57,66^; for a more extensive list, see Lukas and Huchard^54,55^. When committed by males, infanticide is generally associated with male-male sexual competition^51,53,54^, while female-led infanticide tends to occur in the context of intra-group competition for resources or social status^55,61,66^.

Our observation is unlikely to represent a typical case of infanticide for several reasons. First, there was no observation of direct physical harm directed toward the infant during the coalitionary aggression. Second, the individual first observed carrying Rouille after the aggression was a 6-year-old juvenile female (Curtis), followed by her 12-year-old adolescent brother (Cobain), and subsequently by the Kokoalongo adult female Chapman. Infanticide attempts by juveniles are rare in non-human primates^55,67–69^. Third, both Curtis and Chapman exhibited normal carrying behaviour, and Curtis’s behaviour appears typical of juveniles that can occasionally carry 1–2-year-old infants (LF, personal observation). While Cobain, the adolescent male, behaved aggressively toward the infant, his actions more closely resembled those of adolescent bonobos interacting with infrequently targeted prey than unambiguous violent behaviour (MGG, personal observation, Fig. S5). His handling—such as briefly holding the infant by a single extremity without adjusting his grip in response to distress—was characteristic of the careless, non-protective manipulation sometimes observed toward unfamiliar prey and may have caused the wound to the left hand observed on August 7th (Fig. S4).

In many primate species, juveniles and adolescents frequently show strong interest in infants, engaging in behaviours such as touching, carrying, and close inspection, even outside a caregiving context. Such interactions are often interpreted as expressions of curiosity, social learning, attraction to infants, and bond formation, and have been widely documented in non-human primates^70–74^. This broader pattern of juvenile interest in infants may explain why Curtis was the first individual to pick up Rouille after the aggression. Notably, in this instance, Rose did not attempt to approach Curtis to retrieve her infant, which contrasts with the usual behaviour of mothers when in-group members handle their infants. Finally, Chapman, the adult female who ultimately gained access to the infant, cared for and transported Rouille alive for a full day before carrying her corpse, a pattern of behaviour resembling that of chimpanzee and bonobo mothers handling deceased offspring^75–77^(see Fig. S6).

Overall, the behaviour we observed toward the infant did not bear the characteristic aggression of targeted infanticide^78^; this is concordant with the absence of published evidence of infanticide by male or female bonobos. Male aggression toward immature individuals is rare and more frequently targeted at adolescents than infants^78^. The absence of male-led infanticide in bonobos has been attributed to the extended sexual receptivity and cryptic ovulation of female bonobos^79^which obscures knowledge of paternity, reducing the potential fitness benefits of male infanticide^79–85^.

Temporary kidnapping of infants younger than one month has been observed in yellow baboons (*Papio cynocephalus*), Tibetan macaques (*Macaca thibetana*), and Japanese macaques (*Macaca fuscata*) when females are more dominant than the biological mother^86–88^, following severe aggression toward the biological mother by the adopter^86,89^, or following abandonment by their mother^87,88^. Because the details involving the separation of the infant from her mother remain unknown, it is difficult to attribute the behaviours involved to any form of kidnapping. Given that Chapman was only observed carrying Rouille after her two offspring did, we interpret her prolonged carrying as a form of out-group adoption, as Rouille’s mother was not present for relevant care provisioning.

Although out-group adoption is rare among non-human primates, cases have been documented in Angolan black-and-white colobus monkeys (*Colobus angolensis palliatus*)^90^, red howler monkeys (*Alouatta seniculus*)^91^, snub-nosed monkeys (*Rhinopithecus roxellana*)^92^, black-fronted titi monkeys (*Callicebus nigrifrons*)^93^, and bonobos^35^. Unpublished cases of unsuccessful intergroup adoption attempts have also been reported in crested macaques (in Graham et al. 2024)^94^. Notably, both cases of infant adoption by members of other communities of bonobos were successful at Wamba, although the circumstances leading to the initial mother-infant separation remained unknown^35^. Chapman’s attentive care and nursing attempts resembled descriptions of alloparental care observed in adult bonobos and chimpanzees of either sex^35,95^. While alloparental care may have evolved independently in different taxa, it involves neural circuitry attributed to maternal behaviour^96,97^, which is highly conserved across mammals^97–99^. Chapman’s rapid acceptance of Rouille and continuation of care following her death suggest that these mechanisms can be activated quickly, even between unrelated individuals, a finding notable given the single-case nature of this observation.

Several hypotheses have been proposed to explain infant or corpse carrying in primates. In the present case, this observation does not allow a decisive test of these hypotheses; rather, it constrains which explanations are plausible given the absence of prior maternal or affiliative bonds. Frequently cited explanations such as the post-parturient or hormonal hypothesis^100^, learning-to-mother^75,101,102^, and the mother–offspring bond hypothesis^103^are unlikely to apply here, as Chapman was an adoptive female with no previous relationship to Rouille, and Chapman had been a mother of four surviving offspring (Curtis and Cobain, and two females that dispersed previously). By contrast, some hypotheses remain broadly compatible with Chapman’s behaviour, although none provides a fully satisfactory explanation on its own. For instance, the grief management hypothesis^104^proposes that carrying may have stress-buffering benefits, and emotional motivations to care may operate alongside—or override—cognitive aspects of death awareness^100^. Similarly, the unawareness hypothesis^105^suggests that caregivers may continue infant-directed behaviours if death is not fully understood or if the infant is perceived as potentially responsive, which could have contributed to Chapman’s behaviour, given the absence of visible traumatic injuries on Rouille^106,107^.

Additional contextual factors may also have influenced the duration of corpse carrying without fully accounting for it. Rouille’s very young age may have played a role, as reported in Budongo chimpanzees (*Pan troglodytes schweinfurthii*)^77^. Conversely, the rapid decomposition expected under humid conditions may explain Chapman’s abandonment of the corpse after two days, consistent with the slow-decomposition hypothesis^102,108^. Although corpse carrying is often associated with strong mother–infant bonds, such a bond is unlikely here, given the absence of prior affiliation and the individuals’ membership in different social groups—a situation further accentuated by the limited number of EKK–KKL encounters during Rouille’s short lifespan (born June 14, 2024; Fig. S1). Nevertheless, documented cases of out-group adoption by unrelated adults indicate that primates can engage in rapid allomaternal behaviour even in the absence of established social ties^35,90–93^. Taken together, this observation does not fit neatly within existing explanatory frameworks and highlights the need for greater attention to flexible, context-dependent motivational processes underlying corpse-directed behaviour in bonobos.

From an evolutionary perspective, it is noteworthy that Chapman cared for an out-group infant for 24 h and subsequently carried the corpse for two days, despite no detected maternal kinship with Rose (unpublished Kokolopori genetic data; Figure S6). This behaviour parallels reports of biological mothers carrying deceased infants^75,77,106,109^, albeit in a non-kin context. Given bonobos’ high levels of empathy, prosociality, and cooperative tendencies^31,33,44,110–112^, such traits may facilitate spontaneous adoption and caretaking, particularly if mechanisms underlying mother–offspring attachment have been co-opted to support broader social bonding among kin and non-kin^113–115^. Though the precise cause of death remains uncertain, Rouille’s young age and postmortem injuries suggest Cobain’s rough handling may have been pivotal. While infant cannibalism occurs in chimpanzees^10,11,116^, and rarely in bonobos at Kokolopori, it was absent in this case.

Mother-offspring bonds are proposed to underlie the inhibition of maternal cannibalism in chimpanzees^116,117^; thus, although such behaviours are rare and usually associated with high stress^118–120^, Chapman’s restraint here could reflect a significant attachment to Rouille formed over a short period. Additionally, the high food abundance (Fig. S7) and the deteriorating condition of the corpse may have reduced the nutritional incentive to consume it, though this interpretation is based on a single observation. Finally, Rose did not attempt to recover her infant, despite her proximity during Rouille’s vocalisations when Cobain handled her. This may have been influenced by heightened fear following the coalitionary attack that resulted in genital injuries (see Fig. S2) and the threat of retaliation, though we cannot know for sure. It has been proposed that avoidance of retaliation deters attempted recoveries of kidnapped infants^121^. This absence of maternal intervention aligns with the low rates previously reported for bonobo mothers at Kokolopori (relative to Ngogo chimpanzees) and is comparable to the low maternal involvement documented among Gombe chimpanzees (*Pan troglodytes schweinfurthii*^122,123^. Additionally, the rarity of such lethal events may have reduced selective pressures on maternal recovery of infants due to their high risk^121^.

Our observations at Kokolopori highlight the complexity of bonobo social dynamics around intergroup encounters involving individuals from different communities. While bonobos are often regarded as exhibiting less intergroup aggression than chimpanzees and are known for their tolerance and cooperation during intergroup encounters, this incident demonstrates their capacity for coalitionary aggression with potentially fatal—albeit possibly unintended—consequences. These findings challenge oversimplified narratives that strictly divide aggressive chimpanzees and peaceful bonobos, instead pointing to a more nuanced spectrum of intergroup behaviour within the genus *Pan*. This case also illustrates rapid adoption and alloparental care of an out-group infant by a non-kin adult female. Although targeted infanticide in bonobos remains unobserved, the intergroup context and presence of a coordinated out-group coalition may exacerbate coalitionary aggression. Such dynamics offer a plausible pathway through which in-group-out-group biases could lead to fitness-relevant consequences.

## Methods

### Study site and population

All research protocols were approved by the Ministry of Scientific Research of the Democratic Republic of Congo, and the research was carried out in accordance with relevant institutional, national, and international guidelines and regulations (see Ethical note). We conducted daily follows of the habituated bonobo neighbouring communities of Ekalakala and Kokoalongo at the Kokolopori Bonobo Research Site in the Democratic Republic of Congo^124^, which share a total overlapping area of 29.46 km^2^, comprising 62% of Ekalakala’s total home range and 52% of Kokoalongo’s total home range. The non-overlapping portions of the home ranges were 17.9 km^2^ for Ekalakala and 26.46 km^2^ for Kokoalongo^29^. At the time of the documented events, the Ekalakala community consisted of 10 adults (> 15 years old; 8 females and 2 males), and 2 sub-adults (8–14 years old; 1 female and 1 male), and 11 immatures (< 8 years old; 5 females and 6 males), while the KKL community comprised 16 adults (10 females and 6 males), 6 sub-adult males, and 16 immatures (12 females and 4 males) (see Table S1).

### Data collection

#### Behavioural data collection

Behavioural data was collected by a team of researchers, with multiple observers following members of each community. Continuous party composition scans were collected every 30 min. Following the separation of Rose and Rouille, social behaviours involving Rouille were recorded ad libitum. When possible, video data was also recorded ad libitum (video involving Cobain and Rouille can be shared upon request). On August 7th, full-day focal follows were conducted on Rose and Chapman.

## Supplementary Information

Below is the link to the electronic supplementary material.

Supplementary Material 1

Supplementary Material 2

## Data Availability

The datasets generated and/or analysed during the current study are available in the Open Science Framework repository (https://osf.io/b6sxd/).
