# Supplementary material for: A lethal incident during an intergroup encounter in bonobos
Source: Sci Rep. 2026 Mar 23;16:9550. doi: 10.1038/s41598-026-40297-w (PMC13009480; doi:10.1038/s41598-026-40297-w)
Supplement: Supplementary file 1 — Supplementary Material 1 [file 41598_2026_40297_MOESM1_ESM.docx]

**Supplementary Information**

**A lethal incident during an intergroup encounter in bonobos**

Miguel Gareta García; Lillian J. Fornof; Kris H. Sabbi; Floris Martin; Eliana Sonderling; Juliet De Rozario; Mina Cikara; and Martin Surbeck

| **Community** | **Age group** | **Female individuals** | **Male individuals** |
| --- | --- | --- | --- |
| Ekalakala | Immatures  (< 8 y.o) | Verte, Orange, Rouille, Pistache, Vanille | Jade, Bordeaux, Emeraude, Amethyst, Rubin, Avocat |
|  | Sub-adults  (8-14 y.o.) | Acajou | Brun |
|  | Adults  (> 15 y.o.) | Rose, Eben, Olive, Azur, Ivoire, Violette, Bleue | Gris, Noir |
| Kokoalongo | Immatures  (< 8 y.o) | Etta, Micheline, Alicia, Gwen, Tango, Shakira, Enigma, Ozzy, ABBA, Piaf, Curtis, Muse | Prince, Falco, Schubert, Tupac |
|  | Sub-adults  (8-14 y.o.) |  | Freddy, Cobain, Armstrong, Otis, Pop, Toto |
|  | Adults  (> 15 y.o.) | Elliot, PJ, Oliday, Adele, Chapman, Fitz Gloria, Tyler, Dion, Madonna | Fito, Zappa, Bowie, Dylan, Jackson, Guacamole |

**Table S1.** Demographics of the two communities of Ekalakala and Kokoalongo by age groups (infants, juveniles, sub-adults and adults) divided by sex.

| **Community** | **Individual** | **Standardised rank** |
| --- | --- | --- |
| Ekalakala | Noir | 1 |
|  | Violette | 0.923076923 |
|  | Ivoire | 0.846153846 |
|  | Bleue | 0.769230769 |
|  | Eben | 0.692307692 |
|  | Olive | 0.615384615 |
|  | Acajou | 0.538461538 |
|  | Azur | 0.461538462 |
|  | Gris | 0.384615385 |
|  | **Rose** | 0.307692308 |
|  | Jaune | 0.230769231 |
|  | Brun | 0 |
| Kokoalongo | Tyler | 0.956521739 |
|  | Gloria | 0.913043478 |
|  | **Chapman** | 0.869565217 |
|  | Fitz | 0.826086957 |
|  | PJ | 0.782608696 |
|  | Elliot | 0.739130435 |
|  | Oliday | 0.695652174 |
|  | Simone | 0.652173913 |
|  | Guacamole | 0.608695652 |
|  | Toto | 0.565217391 |
|  | Adele | 0.52173913 |
|  | Dylan | 0.47826087 |
|  | Madonna | 0.434782609 |
|  | Zappa | 0.391304348 |
|  | Cobain | 0.347826087 |
|  | Bowie | 0.260869565 |
|  | Otis | 0.173913043 |
|  | Jackson | 0.130434783 |
|  | Pop | 0.086956522 |
|  | Freddy | 0.043478261 |
|  | Fito | 0 |

**Table S2.** Table S2. Standardised dominance ranks for adults and sub‑adults with available aggression data from 2024 in the Ekalakala and Kokoalongo communities. Ranks were derived from within‑community aggression interactions with a clear winner and loser, using the Elo‑rating package in R [1]. Values close to 1 indicate high rank, whereas values near 0 indicate low rank.

**
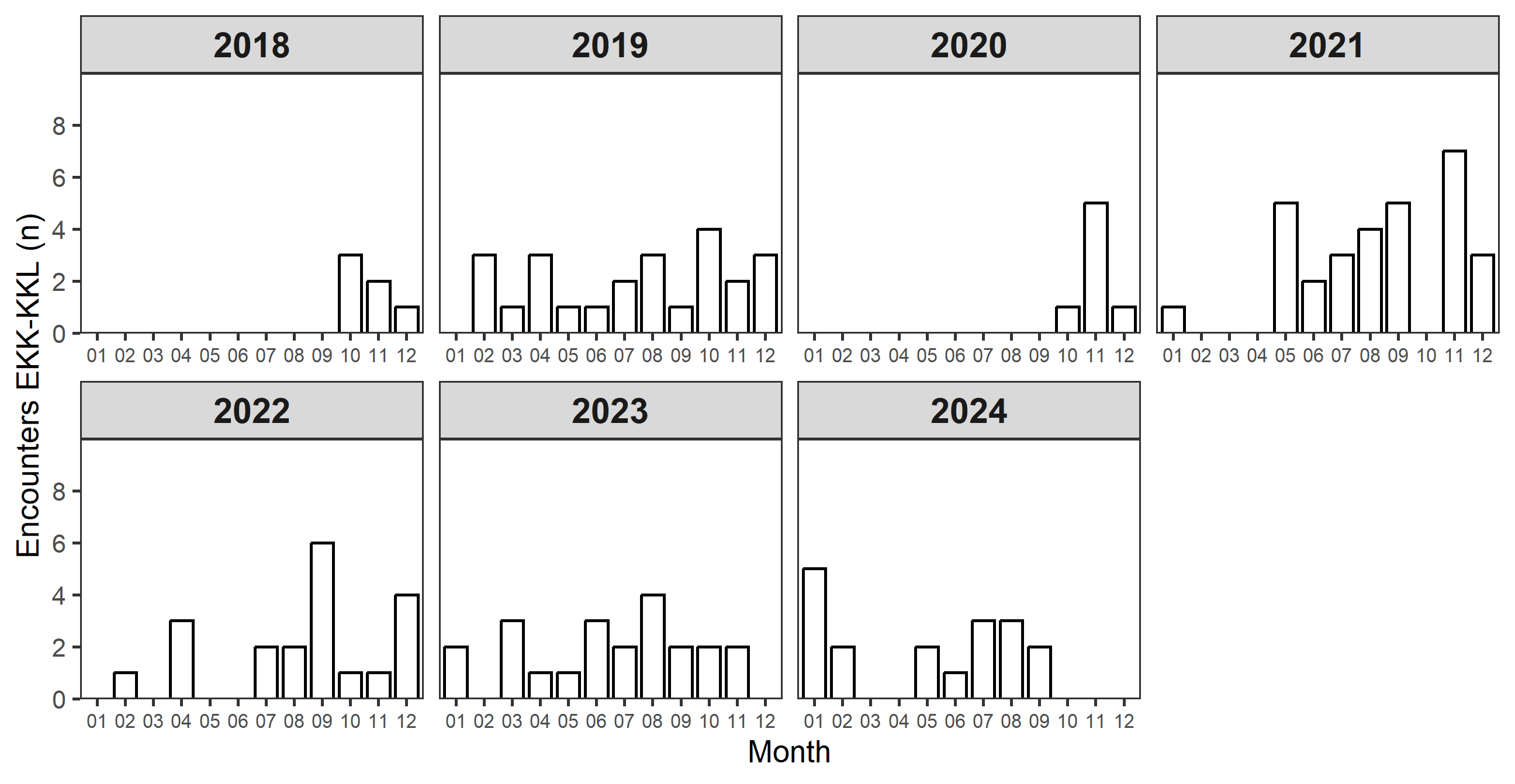
Figure S1.** Intergroup encounters between Ekalakala (EKK) and Kokoalongo (KKL) from October 2018 to September 2024. Months are shown on the x-axis in ascending order, and the number of encounters appears on the y-axis. Encounters are considered distinct when separated by at least 24 hours. An encounter is included only when at least two adults or subadults from one community are observed mingling with individuals from the other community. Some months with no recorded encounters correspond either to periods without systematic observations (January to September 2018) or to interruptions in the project due to COVID-19 restrictions (March-August 2020).

| **Year** | **Mean duration** | **S.D. duration** | **Maximum duration** | **Minimum duration** | **Number of distinct encounters** |
| --- | --- | --- | --- | --- | --- |
| 2018 | 1.727 | 1.009 | 4 | 1 | 6 |
| 2019 | 5.027 | 4.204 | 17 | 1 | 24 |
| 2020 | 1.222 | 0.440 | 2 | 1 | 7 |
| 2021 | 5.267 | 3.838 | 15 | 1 | 30 |
| 2022 | 4.419 | 3.446 | 16 | 1 | 20 |
| 2023 | 8.771 | 8.173 | 36 | 1 | 22 |
| 2024 | 6.510 | 5.826 | 23 | 1 | 18 |

**Table S3.** Mean duration, standard deviation, maximum, and minimum duration of intergroup encounters between the Ekalakala (EKK) and Kokoalongo (KKL) communities across years (October 2018–September 2024). An encounter is defined as a period during which mixed-group party compositions occurred and is considered continuous if subsequent mixed-group associations reoccurred within 24 h; mixed-group associations separated by ≥24 h were classified as independent encounters. Encounter duration is expressed as the number of 30-min observation intervals in which mixed-group party composition was recorded within each **
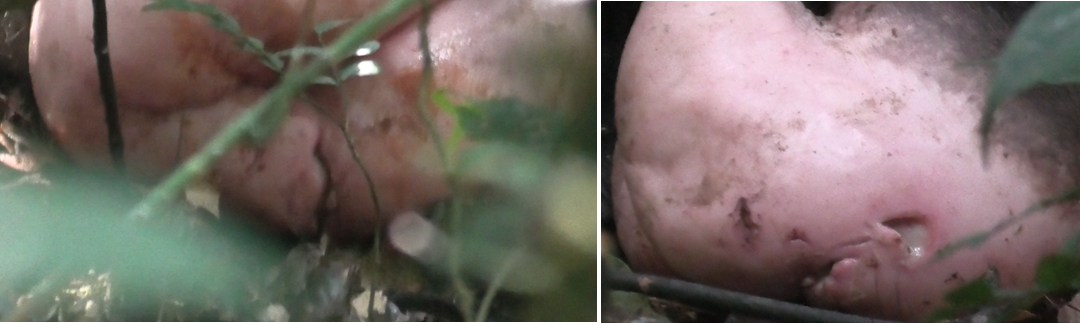
**encounter. The rightmost column reports the number of independent encounters per year.

**Figure S2.** Photographs (by FM) of Rose’s genital injuries taken on August 11, 2024. Given the absence of any other aggressions in the days following the Kokoalongo coalitionary attack, these wounds were likely caused during that incident.


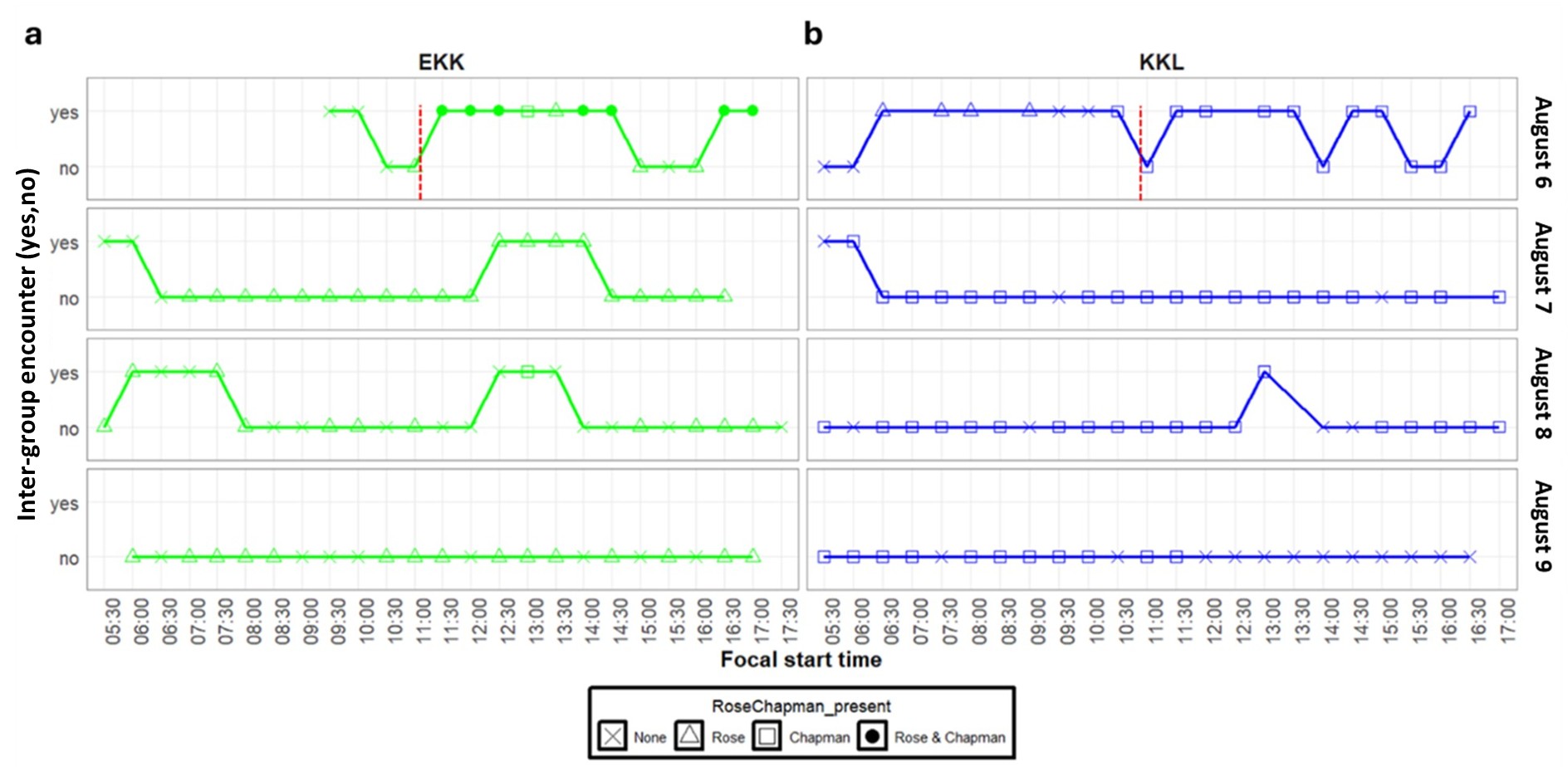


**Figure S3.** Party composition data were collected by two separate observers every half hour.

a) The left (green) graph shows party compositions recorded by an observer following the Ekalakala community (EKK), while b) the right graph (blue) shows those from an observer following the Kokoalongo community (KKL). The y-axis indicates whether individuals from the other community were present during the inter-group encounter (yes/no). The plots represent consecutive days from top to bottom. The adoption occurred on August 6 at approximately 11:00 a.m. (red dotted line), and Rouille’s corpse was abandoned on August 9. Symbols represent key individuals: (i) Rose (biological mother, EKK) as empty triangles, (ii) Chapman (adoptive mother, KKL) as empty squares, (iii) both Rose and Chapman as full circles, and (iv) neither as a cross.

| **Date** | **Community** | **Hour** | **Party composition** | **Intergroup Encounter context** | **Rose**  **present** | **Chapman**  **present** | **Rose & Chapman**  **present** |
| --- | --- | --- | --- | --- | --- | --- | --- |
| 06/08/2024 | EKK | 10:00 | **Bowie**;Eben;Jaune;**Madonna**;Noir;Violette;**Zappa** | yes | no | no | no |
| 06/08/2024 | EKK | 10:30 | Azur;Bleue;Brun;Celeste;Gris;Ivoire;Jaune;Pistache;UNK;Vanille;Violette | no | no | no | no |
| 06/08/2024 | EKK | 11:00 | Azur;Bleue;Brun;Celeste;Eben;Gris;Ivoire;Jaune;Olive;Pistache;Rose;Saphir;Violette;Vanille | no | yes | no | no |
| 06/08/2024 | EKK | 11:30 | Azur;Bleue;Brun;Celeste;**Chapma**n;Eben;**Fitz**;Gris;Jaune;**Madonna**;Olive;Pistache;Rose;UNK | yes | yes | yes | yes |
| 06/08/2024 | EKK | 12:00 | Bleue;**Bowie**;Brun;**Chapman**;Eben;**Elliot;Gloria**;Gris;Ivoire;**Madonna**;Noir;**Oliday**;Pistache;**PJ**;Pop;Rose;**Schubert;Tupac;Tyler**;Vanille;Violette;**Zappa** | yes | yes | yes | yes |
| 06/08/2024 | EKK | 12:30 | **Bowie**;Brun;**Chapman**;Eben;**Fitz;Gloria**;Gris;Ivoire;Jaune;**Madonna**;Noir;Olive;Pistache;Pop;Rose;Saphir**;Schubert;Tupac;Tyler**;Violette | yes | yes | yes | yes |
| 06/08/2024 | EKK | 13:00 | Azur;Bleue;**Bowie**;**Chapman**;**Elliot;Gloria**;Gris;Ivoire;Noir;Olive;Pistache;**PJ;Pop;Schubert;Toto;Tupac;Tyler**;UNK;**Willow;Zappa** | yes | no | yes | no |
| 06/08/2024 | EKK | 13:30 | Brun;Gris;Ivoire;Jaune;Noir;**Oliday**;Olive;Pistache;**Pop**;Rose;**Tupac**;Vanille | yes | yes | no | no |
| 06/08/2024 | EKK | 14:00 | Azur;Bleue**;Bowie**;Brun;Celeste;**Chapman**;**Cobain;Elliot;Fitz;Gloria**;Gris;**Guacamole**;Ivoire;**Madonna;Nancy**;Noir;Olive;**Ozzy**;Pistache;**PJ;Pop**;Rose;Saphir;**Schubert;Simone;Toto;Tupac;Tyler**;UNK;Violette;**Zappa**;Vanille | yes | yes | yes | yes |
| 06/08/2024 | EKK | 14:30 | Bleue;**Bowie**;Brun;Celeste;**Chapman**;**Cobain;Fitz**;Gris;**Guacamole**;Ivoire;**Madonna;Nancy;Oliday**;Pistache;**PJ**;Rose;Saphir;**Schubert;Simone;Toto;Tupac;Tyler**;Violette;Willow;**Zappa** | yes | yes | yes | yes |
| 06/08/2024 | EKK | 15:00 | Azur;Bleue;Brun;Celeste;Eben;Ivoire;Rose;Saphir;Vanille;Violette | no | yes | no | no |
| 06/08/2024 | EKK | 15:30 | Azur;Bleue;Brun;Celeste;Eben;Gris;Ivoire;Jaune;Noir;Olive;Pistache;Saphir;Vanille;Violette | no | no | no | no |
| 06/08/2024 | EKK | 16:00 | Azur;Bleue;Brun;Celeste;Eben;Ivoire;Jaune;Noir;Rose;Violette | no | yes | no | no |
| 06/08/2024 | EKK | 16:30 | Azur;Bleue;**Bowie**;Brun;Celeste;**Chapman**;**Cobain;Eben;Elliot;Fitz;Gloria**;Gris;**Guacamole**;Ivoire;Jaune;**Madonna;Nancy**;Noir;**Oliday**;Olive;**Otis**;Pistache;**PJ;Pop**;Rose;**Saphir;Schubert;Simone;Toto;Tupac;Tyler**;Vanille;Violette;**Zappa** | yes | yes | yes | yes |
| 06/08/2024 | EKK | 17:00 | Acajou;Azur;Bleue;**Bowie**;Brun;Celeste;**Chapman**;**Cobain**;Eben;**Elliot;Fitz;Gloria**;Gris;**Guacamole**;Ivoire;Jaune;**Madonna;Nancy**;Noir;**Oliday**;Olive;**Otis**;Pistache;**PJ;Pop**;Rose;Saphir;**Schubert;Simone;Toto;Tupac;Tyle**r;UNK;Vanille;Violette;**Zappa** | yes | yes | yes | yes |
| 06/08/2024 | KKL | 05:30 | Bowie;Fitz;Freddy;Gloria;Madonna;Willow;Zappa | no | no | no | no |
| 06/08/2024 | KKL | 06:00 | Bowie;Elliot;Fitz;Freddy;Gloria;Madonna;Nancy;Willow;Zappa | no | no | no | no |
| 06/08/2024 | KKL | 06:30 | **Azur;Bleue**;Bowie;**Brun**;Elliot;Fitz;Freddy;Gloria;**Ivoire**;Madonna;**Noir;Pistache**;**Rose**;**Vanille;Violette**;Zappa | yes | yes | no | no |
| 06/08/2024 | KKL | 07:30 | **Azur;Bleue**;Bowie;**Brun;Celeste**;Elliot;Fitz;Freddy;Gloria;**Ivoire**;Madonna;**Noir**;**Rose**;**Violette**;Zappa | yes | yes | no | no |
| 06/08/2024 | KKL | 08:00 | **Azur;Bleue**;Bowie;**Brun;Celeste**;Elliot;Fitz;Freddy;**Gris;Ivoire**;Madonna;Nancy;**Noir**;**Rose**;**Violette**;Willow;**Zappa** | yes | yes | no | no |
| 06/08/2024 | KKL | 09:00 | **Azur;Bleue**;Bowie;**Brun;Celeste**;Elliot;Fitz;Freddy;Gloria;**Ivoire**;Madonna;Nancy;**Noir;Pistache**;**Rose**;Toto;**Violette**;Willow;Zappa | yes | yes | no | no |
| 06/08/2024 | KKL | 09:30 | **Bleue;Celeste**;Fitz;Freddy;Gloria;Gris;Ivoire;Nancy;**Noir;Pistache;Violette**;Willow;Zappa | yes | no | no | no |
| 06/08/2024 | KKL | 10:00 | **Azur;Bleue;Brun**;Elliot;Freddy;Gloria;**Gris;Ivoire;Noir;Pistache;Violette**;Zappa | yes | no | no | no |
| 06/08/2024 | KKL | 10:30 | **Azur;Bleue**;Bowie;**Brun;Celeste**;**Chapman**;Cobain;Elliot;Fitz;Freddy;Gloria;**Gris**;Guacamole;**Ivoire**;Madonna;Nancy;**Noir**;Oliday;Otis;Ozzy;**Pistache**;PJ;Pop;Schubert;Simone;Toto;Tupac;Tyler;**Violette**;Willow;Zappa | yes | no | yes | no |
| 06/08/2024 | KKL | 11:00 | Bowie;**Chapman**;Cobain;Elliot;Freddy;Gloria;Guacamole;Madonna;Oliday;Otis;PJ;Schubert;Simone;Toto;Tupac;Tyler;Zappa | no | no | yes | no |
| 06/08/2024 | KKL | 11:30 | **Brun**;Chapman;Cobain;Freddy;**Gris;Ivoire**;Madonna;**Noir;Pistache**;PJ;Pop;Schubert;Simone;Toto;**Violette** | yes | no | yes | no |
| 06/08/2024 | KKL | 12:00 | **Azur**;Bowie;Brun;Chapman;Cobain;Fitz;Gloria;Guacamole;**Ivoire**;Ozzy;**Pistache**;PJ;Pop;Tupac;Tyler;**Violette**;Zappa | yes | no | yes | no |
| 06/08/2024 | KKL | 13:00 | **Brun**;Chapman;Cobain;Freddy;Guacamole;Madonna;PJ;Schubert;Simone;**Violette**;Zappa | yes | no | yes | no |
| 06/08/2024 | KKL | 13:30 | **Azur;Brun**;Chapman;Cobain;Elliot;Freddy;Guacamole;**Ivoire**;Madonna;**Noir;Pistache**;Pop;Schubert;Simone;Toto;Tyler;**Violette**;Zappa | yes | no | yes | no |
| 06/08/2024 | KKL | 14:00 | Bowie;Chapman;Cobain;Fitz;Madonna;Zappa | no | no | yes | no |
| 06/08/2024 | KKL | 14:30 | **Bleue**;Bowie;**Brun**;Chapman;Cobain;**Eben**;Fitz;Gloria;Guacamole;**Ivoire;Pistache**;PJ;Pop;Schubert;Toto;Tyler;**Violette**;Zappa | yes | no | yes | no |
| 06/08/2024 | KKL | 15:00 | Chapman;Cobain;Elliot;Fitz;Freddy;Gloria;**Gris**;Guacamole;**Ivoire**;Madonna;Otis;PJ;Pop;Schubert;Simone;Toto;Tupac;Tyler;Zappa | yes | no | yes | no |
| 06/08/2024 | KKL | 15:30 | Bowie;Chapman;Cobain;Elliot;Fitz;Freddy;Guacamole;Madonna;Ozzy;PJ;Pop;Schubert;Simone;Tupac;Tyler;Zappa | no | no | yes | no |
| 06/08/2024 | KKL | 16:00 | Bowie;Chapman;Cobain;Elliot;Fitz;Freddy;Gloria;Guacamole;Madonna;Nancy;Oliday;Otis;Ozzy;PJ;Pop;Schubert;Simone;Toto;Tupac;Tyler;Willow;Zappa | no | no | yes | no |
| 06/08/2024 | KKL | 16:30 | **Azur;**Bowie;**Brun**;Chapman;Cobain;Fitz;Freddy;Gloria;**Gris**;Guacamole;**Ivoire**;Madonna;**Noir**;Oliday;Otis;PJ;Pop;Schubert;Simone;Toto;Tupac;Tyler;**Violette**;Zappa | yes | no | yes | no |
| 07/08/2024 | EKK | 05:30 | Bleue;**Bowie;Simone;Tyler** | yes | no | no | no |
| 07/08/2024 | EKK | 06:00 | Bleue;**Cobain;Elliot;Freddy;Gloria**;Gris;Ivoire;**Madonna**;Noir;**Simone;Tyler** | yes | no | no | no |
| 07/08/2024 | EKK | 06:30 | Azur;Bleue;Brun;Gris;Ivoire;Jaune;Noir;Olive;Saphir;Violette | no | no | no | no |
| 07/08/2024 | EKK | 07:00 | Azur;Bleue;Brun;Celeste;Gris;Ivoire;Jaune;Noir;Olive;Pistache;Rose;Saphir;Violette | no | yes | no | no |
| 07/08/2024 | EKK | 07:30 | Azur;Bleue;Brun;Celeste;Eben;Ivoire;Jaune;Noir;Olive;Pistache;Rose;Saphir;Violette | no | yes | no | no |
| 07/08/2024 | EKK | 08:00 | Bleue;Brun;Jaune;Noir;Olive;Rose;Saphir | no | yes | no | no |
| 07/08/2024 | EKK | 08:30 | Azur;Rose;Saphir;Violette | no | yes | no | no |
| 07/08/2024 | EKK | 09:00 | Azur;Rose;Saphir;Violette | no | yes | no | no |
| 07/08/2024 | EKK | 09:30 | Azur;Bleue;Jaune;Olive;Pistache;Rose;Saphir;Violette | no | yes | no | no |
| 07/08/2024 | EKK | 10:00 | Azur;Rose;Violette | no | yes | no | no |
| 07/08/2024 | EKK | 10:30 | Azur;Bleue;Olive;Rose;Violette | no | yes | no | no |
| 07/08/2024 | EKK | 11:00 | Azur;Bleue;Brun;Eben;Ivoire;Noir;Olive;Rose;Saphir;Violette | no | yes | no | no |
| 07/08/2024 | EKK | 11:30 | Azur;Bleue;Brun;Eben;Gris;Ivoire;Jaune;Noir;Olive;Pistache;Rose;Saphir;Violette | no | yes | no | no |
| 07/08/2024 | EKK | 12:00 | Azur;Bleue;Brun;Gris;Jaune;Rose;Saphir;Violette | no | yes | no | no |
| 07/08/2024 | EKK | 12:30 | Brun;Olive;Rose;**Toto;Tyler;Violette;Willow** | yes | yes | no | no |
| 07/08/2024 | EKK | 13:00 | Azur;Bleue;Gris;Ivoire;Noir;Olive;Rose;Saphir;**Toto;Tyler;Violette;Willow** | yes | yes | no | no |
| 07/08/2024 | EKK | 13:30 | Azur;Bleue;Brun;Celeste;Gris;**Guacamole**;Ivoire;Noir;Olive;Rose;**Toto;Tupac;Tyler** | yes | yes | no | no |
| 07/08/2024 | EKK | 14:00 | Azur;Bleue;Brun;Celeste;Gris;**Guacamole**;Ivoire;Jaune;**Nancy**;Noir;**Oliday**;Olive;Pistache;**PJ;**Rose;Saphir;**Schubert;Simone;Toto;Tupac;Tyler**;Violette | yes | yes | no | no |
| 07/08/2024 | EKK | 14:30 | Azur;Bleue;Brun;Eben;Gris;Ivoire;Noir;Olive;Pistache;Rose;Violette | no | yes | no | no |
| 07/08/2024 | EKK | 15:00 | Azur;Bleue;Brun;Celeste;Eben;Gris;Ivoire;Jaune;Noir;Olive;Pistache;Rose;Saphir;Violette | no | yes | no | no |
| 07/08/2024 | EKK | 15:30 | Azur;Bleue;Brun;Celeste;Eben;Ivoire;Jaune;Noir;Olive;Pistache;Rose;Saphir;Violette | no | yes | no | no |
| 07/08/2024 | EKK | 16:00 | Azur;Bleue;Celeste;Ivoire;Jaune;Noir;Olive;Pistache;Rose;Saphir;Violette | no | yes | no | no |
| 07/08/2024 | EKK | 16:30 | Azur;Bleue;Brun;Celeste;Eben;Gris;Ivoire;Jaune;Noir;Olive;Pistache;Rose;Saphir;Violette | no | yes | no | no |
| 07/08/2024 | KKL | 05:30 | **Bleue**;Bowie;Cobain;Toto;Tyler | yes | no | no | no |
| 07/08/2024 | KKL | 06:00 | **Azur;Bleue**;Bowie;Chapman;Cobain;Elliot;Freddy;Gloria;**Gris;Ivoire**;Madonna;**Noir**;PJ;Toto;Tyler;Zappa | yes | no | yes | no |
| 07/08/2024 | KKL | 06:30 | Bowie;Chapman;Cobain;Elliot;Freddy;Gloria;Guacamole;Oliday;Simone;Toto;Tupac;Zappa | no | no | yes | no |
| 07/08/2024 | KKL | 07:00 | Bowie;Chapman;Cobain;Elliot;Fitz;Freddy;Gloria;Guacamole;Madonna;PJ;Pop;Toto;Tupac;Tyler;Zappa | no | no | yes | no |
| 07/08/2024 | KKL | 07:30 | Chapman;Cobain;Nancy;PJ;Schubert;Simone;Toto;Tupac;Tyler | no | no | yes | no |
| 07/08/2024 | KKL | 08:00 | Bowie;Chapman;Cobain;Fitz;Freddy;Gloria;Guacamole;PJ;Schubert;Simone;Toto;Tupac;Tyler;Zappa | no | no | yes | no |
| 07/08/2024 | KKL | 08:30 | Bowie;Chapman;Cobain;Elliot;Fitz;Freddy;Gloria;Guacamole;Madonna;Nancy;Oliday;PJ;Pop;Schubert;Simone;Toto;Zappa | no | no | yes | no |
| 07/08/2024 | KKL | 09:00 | Bowie;Chapman;Cobain;Freddy;Guacamole;Nancy;Pop;Schubert;Tyler;Willow;Zappa | no | no | yes | no |
| 07/08/2024 | KKL | 09:30 | Bowie;Cobain;Elliot;Fitz;Freddy;Guacamole;Madonna;Nancy;Pop;Toto;Tupac;Tyler;Willow;Zappa | no | no | no | no |
| 07/08/2024 | KKL | 10:00 | Bowie;Chapman;Cobain;Elliot;Fitz;Freddy;Gloria;Guacamole;Madonna;Nancy;PJ;Pop;Schubert;Simone;Toto;Tupac;Tyler;Willow;Zappa | no | no | yes | no |
| 07/08/2024 | KKL | 10:30 | Bowie;Chapman;Cobain;Elliot;Gloria;Guacamole;Madonna;Nancy;Oliday;PJ;Pop;Schubert;Toto;Tupac;Tyler;Zappa | no | no | yes | no |
| 07/08/2024 | KKL | 11:00 | Bowie;Chapman;Cobain;Guacamole;Madonna;Nancy;Oliday;Ozzy;PJ;Pop;Schubert;Simone;Toto;Tupac;Tyler;Zappa | no | no | yes | no |
| 07/08/2024 | KKL | 11:30 | Chapman;Cobain;Fitz;Freddy;Gloria;Guacamole;Madonna;Schubert | no | no | yes | no |
| 07/08/2024 | KKL | 12:00 | Bowie;Chapman;Cobain;Fitz;Freddy;Madonna;Zappa | no | no | yes | no |
| 07/08/2024 | KKL | 12:30 | Chapman | no | no | yes | no |
| 07/08/2024 | KKL | 13:00 | Chapman;Fitz;Madonna | no | no | yes | no |
| 07/08/2024 | KKL | 13:30 | Bowie;Chapman;Fitz;Madonna | no | no | yes | no |
| 07/08/2024 | KKL | 14:00 | Bowie;Chapman;Cobain;Fitz;Freddy;Madonna;Zappa | no | no | yes | no |
| 07/08/2024 | KKL | 14:30 | Bowie;Chapman;Cobain;Fitz;Freddy;Gloria;Guacamole;Madonna;Nancy;Oliday;Otis;Ozzy;PJ;Pop;Schubert;Simone;Toto;Tupac;Tyler;Willow;Zappa | no | no | yes | no |
| 07/08/2024 | KKL | 15:00 | Guacamole;Nancy;Oliday;PJ;Pop;Schubert;Simone;Toto;Tupac;Tyler;Willow;Zappa | no | no | no | no |
| 07/08/2024 | KKL | 15:30 | Bowie;Chapman;Elliot;Fitz;Freddy;Gloria;Guacamole;Oliday;Ozzy;Schubert;Simone;Tyler;Willow;Zappa | no | no | yes | no |
| 07/08/2024 | KKL | 16:00 | Chapman;Cobain;Elliot;Gloria;Guacamole;Nancy;PJ;Pop;Schubert;Simone;Toto;Tupac;Tyler;Willow;Zappa | no | no | yes | no |
| 07/08/2024 | KKL | 17:00 | Bowie;Chapman;Elliot;Freddy;Gloria;Guacamole;Madonna;Nancy;Ozzy;PJ;Pop;Schubert;Toto;Tupac;Tyler;Willow;Zappa | no | no | yes | no |

**Table S4**. Party composition scans were collected on August 6^th^ and 7^th^ in the Ekalakala (EKK) and Kokoalongo (KKL) communities in encounter and non-encounter conditions. Names in bold are out-group individuals mingling with the community that the observer was following. The community refers to the group that the observer is following and the time of the 30-minute party scan during which party composition was recorded. Chapman and Rose appear highlighted in red and green, respectively, and in bold when they were observed mingling within a party composition of another group, thus considered outgroup individuals. Columns to the right indicate whether inter-group encounter conditions were met or not. The columns Rose present, Chapman present, or Rose & Chapman present indicate whether the individuals implied were observed in the party scans. UNK refers to cases when the researcher was unable to identify the individual.

**
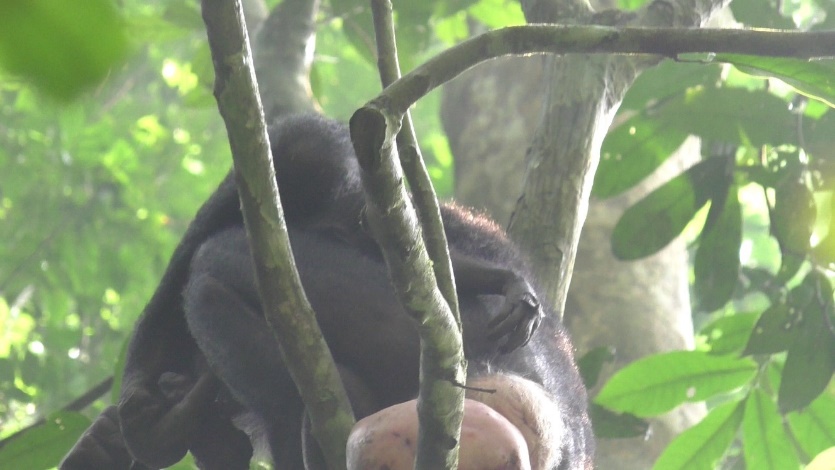
**

**Figure S4**. Rouille on Chapman’s abdomen while she’s foraging on a tree on August 7, 2025. An open wound is visible on Rouille’s right hand.


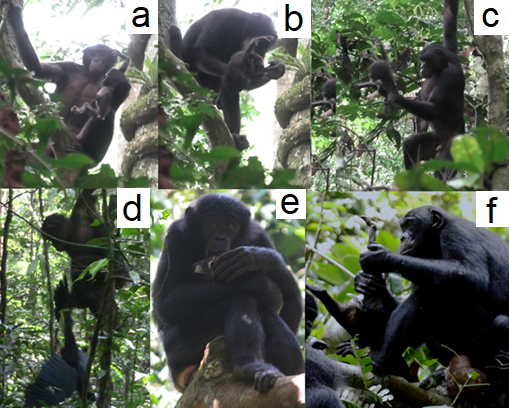


**Figure S5**. Photos showing various interactions of bonobos. (a–c) Cobain handling Rouille on August 6, 2025 (photo: FM). (d) Adolescent KKL individual Tupac holding a live turaco upside down, which he found, transported while climbing trees, and later dragged along the ground on June 19, 2024 (MGG). (e, f) Dispersing subadult female Saphir (e) holding and biting a duiker hunted by the EKK community. (f) Adult female Bleue holding the same duiker upside down and repeatedly biting a duiker hunted by the EKK community. The duiker did not suffer severe injuries nor die, and was released by the bonobos, who had abandoned it after multiple individuals had handled it (February 14, 2022, MGG).


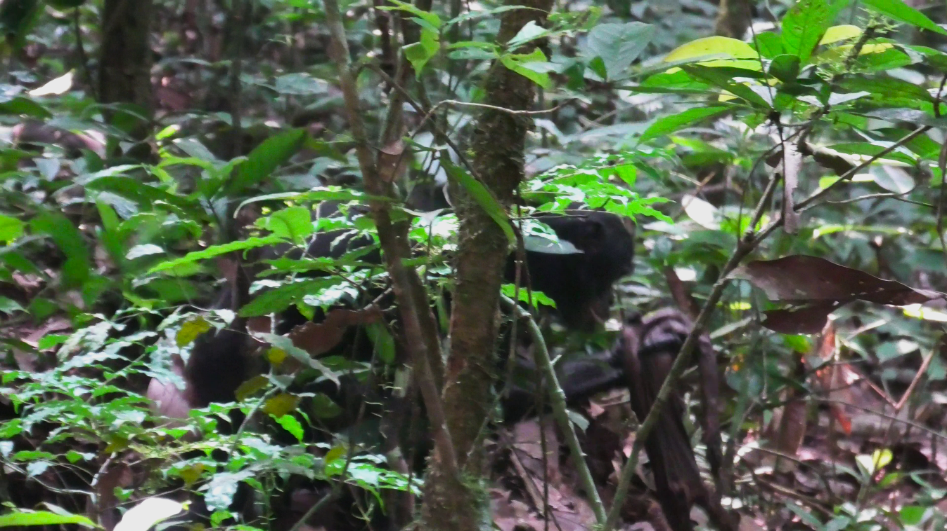


**Figure S6.** Chapman was carrying Rouille’s corpse with her left hand on the morning of August 9, 2024, shortly before she finally dropped it.


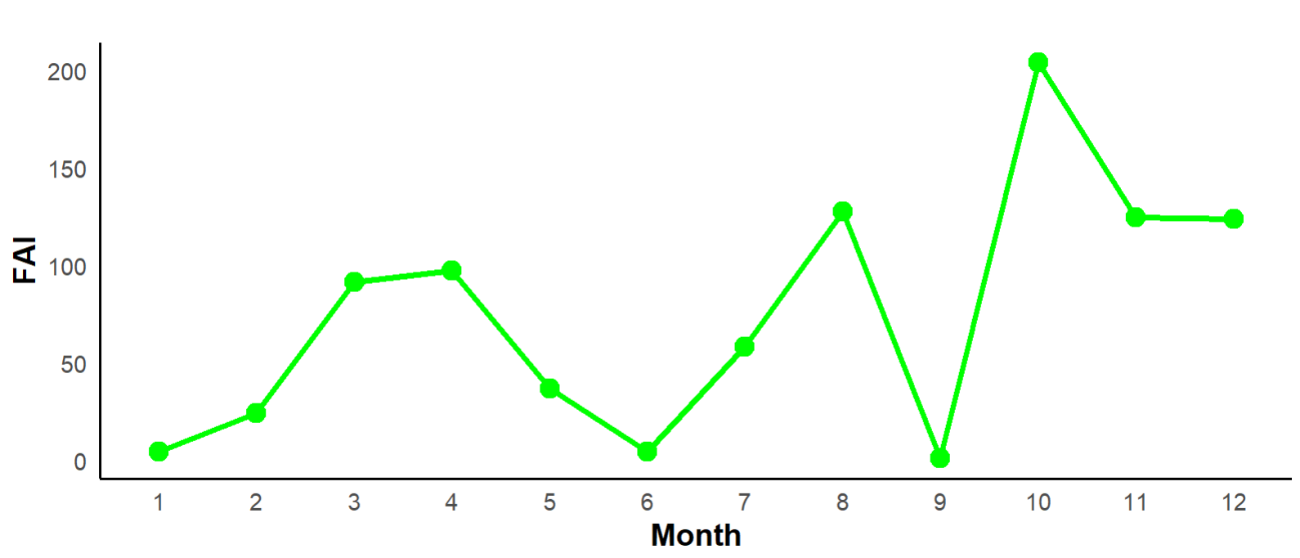


**Figure S7.** Food abundance index as a proxy of food availability [2–4]. The months corresponding to the year 2024 are represented along the x-axis, and the food abundance along the y-axis. We calculated a Monthly Food Availability Index (MFAI) by combining data on monthly consumption of fruits of tree and vine species from the focal follows, fruiting data from phenology transects, and vegetation plot surveys. In the equation below:

_S_

MFAI = ∑*P_im_ * B_i_*

*P_im_* represents the proportion of trees from the phenology transects bearing fruit each month *m,* and *Bi* represents the cross-sectional basal area of the species *i* (at 1.3m above ground level and gathered in plot data on each tree species) and *S* is the total number of species in the monthly bonobo diet.

**References**

1. Neumann, C. *et al.* Assessing dominance hierarchies: validation and advantages of progressive evaluation with Elo-rating. *Anim Behav* **82**, 911–921 (2011).

2. Wessling, E. G. *et al.* Seasonal variation in physiology challenges the notion of Chimpanzees (Pan troglodytes verus) as a forest-adapted species. *Front Ecol Evol* **6**, (2018).

3. Wessling, E. G., Kühl, H. S., Mundry, R., Deschner, T. & Pruetz, J. D. The costs of living at the edge: Seasonal stress in wild savanna-dwelling chimpanzees. *J Hum Evol* **121**, 1–11 (2018).

4. Lucchesi, S. *et al.* Beyond the group: how food, mates, and group size influence intergroup encounters in wild bonobos. *Beheavioral Ecology* 1–14 (2020) doi:10.1093/beheco/arz214.
